# Valproic-induced Fanconi syndrome: Clinical features, risk factors, diagnosis and management

**DOI:** 10.3389/fmed.2022.945244

**Published:** 2022-09-16

**Authors:** Chunjiang Wang, Yulu Zhou, Liying Song, Zhenzhen Deng, Weijin Fang

**Affiliations:** ^1^Department of Pharmacy, The Third Xiangya Hospital, Central South University, Changsha, Hunan, China; ^2^Department of Pharmacy, Hunan Provincial Maternal and Child Health Care Hospital, Changsha, Hunan, China

**Keywords:** valproic acid, Fanconi syndrome, epilepsy, kidney disease, carnitine

## Abstract

**Objective:**

Although Fanconi syndrome (FS) induced by valproate (VPA) has occasionally been reported, the detailed clinical features of the disease remain unclear. The aim of this study was to elucidate the clinical features of patients with VPA-induced FS.

**Methods:**

We searched Chinese and English databases for all original studies, clinical reports, and case reports on VPA-induced FS published before March 2022.

**Results:**

A total of 29 articles including 54 patients (28 males and 24 females) were included. The patients had a median age of 7 years (range 2–34 years), had severely disabled (87.0%), tube feeding (64.8%), and received an average of 1.8 medications other than VPA. The median duration of VPA treatment was 4 years (range 0.7–15.5). Pathological fractures (25.9%), unexplained fever (11.1%), muscle weakness (9.3%), and edema (9.3%) were the most common symptoms, while 18 patients were diagnosed in incidental laboratory tests. Blood tests revealed hypokalemia (69.2%), hypophosphatemia (98.0%), and hypouricemia (93.3%). Urinalysis revealed glucosuria (96.1%), proteinuria (100.0%), generalized hyperaminoaciduria (100.0 %), β2 macroglobulin (100.0%). Decreased percent total reabsorption of phosphate (%TRP) found in 94.1% of patients, and increased fractional excretion of uric acid (FEUA) were found in 100% of patients. The median time to resolution of FS after discontinuation of drug therapy was 3 months (range 0.25–18).

**Conclusions:**

The possibility of FS needs to be considered with long-term VPA administration, especially in young, tube-fed, severely disabled patients who are co-administered with anticonvulsants. Patients receiving VPA should have regular blood and urine tests. Abnormal laboratory values returned to normal levels after VPA discontinuation.

## Introduction

Fanconi syndrome (FS) is a systemic proximal tubular dysfunction characterized by proteinuria, glycosuria, phosphateuria, generalized hyperaminoaciduria, hypokalemia, hypophosphatemia, and type II renal tubular acidosis (1). Fanconi syndrome can be triggered by different causes, including hereditary, idiopathic, and acquired. Drugs are the most common cause of acquired FS, including aminoglycosides, various chemotherapeutics, antiepileptic drugs, and iron chelators (2).

Valproic acid (VPA) is a potent antiepileptic drug (AED) for generalized and partial epilepsy in adults and children (3, 4). Adverse effects of VPA include gastrointestinal disturbances, elevated liver enzymes, hyperammonemia, neurological disturbances, alopecia, weight gain, pancreatitis, and thrombocytopenia (5). Renal involvement is an uncommon adverse effect of VPA (6–9), and VPA-induced FS is rarely reported. This complication was first reported by Lenoir et al. (10). The understanding of VPA induced FS is mainly based on case reports. While the detailed clinical and laboratory features and exact pathogenic mechanisms of VPA-induced FS remain unclear and are easily overlooked in clinical practice, leading to delayed diagnosis and treatment. The purpose of this study was to discuss the clinical features, diagnosis, pathogenesis, and management of VPA-induced FS.

## Methods

### Search strategy and data sources

We searched for all original research studies, clinical reports, case reports and reviews of valproic acid-related renal toxicity published before March 2022 in the PubMed, EMBASE, Wanfang and CNKI databases. Search using a combination of subject words and free words, including valproate, sodium valproate, valproic acid, antiepileptic drugs, Fanconi syndrome, DeToni-Debre-Fanconi syndrome, tubulointerstitial nephritis, renal tubular acidosis. Inclusion criteria were as follows: (1) human subjects; (2) papers published online; (3) case reports should have detailed medical history, laboratory tests, treatment or prognosis.

### Data extraction

We extracted author, year, age, gender; medical history, dosage, route of administration, duration, blood concentrations, carnitine levels, clinical characteristics, laboratory tests, renal biopsies, treatment, and prognosis using self-designed tables. Laboratory data include blood gas analysis, serum potassium, serum uric acid, creatinine, urea nitrogen, phosphate and carnitine, as well as urine pH, protein, glucose, uric acid and β2 macroglobulin (β2-MG), fractional excretion of uric acid (FEUA), fractional excretion of potassium (FEK), fractional excretion of sodium (FENa) and percent total reabsorption of phosphate (%TRP).

### Classification of Fanconi syndrome

Complete Fanconi syndrome is defined as systemic dysfunction of the proximal renal tubules resulting in excessive urinary excretion of amino acids, glucose, phosphate, bicarbonate, and protein. Incomplete Fanconi syndrome is defined as partial proximal renal tubular dysfunction resulting in excessive excretion of some of these solutes in the urine (11).

### Statistical analysis

Descriptive analysis was performed on the extracted data. The enumeration data were expressed by the number of cases and percentage. The measurement data is represented by the median value (minimum, maximum).

## Results

### Our sample

A total of 129 publications were screened, and after eligibility assessment of these articles, 29 articles were finally included in the study. We identified 54 patients (28 males and 24 females) with a median age of 7 years (range 2–34 years) (Table 1). These patients were mainly from Asia (29 cases), Europe (15 cases), and North America (1 case). Of the 54 patients identified, 47 patients (87.0%) were described as severely disabled patients (bed-ridden and fed by a nasogastric or gastrostomy tube). Feeding was described in 38 of 54 patients, of whom 35 patients (64.8%) reported tube feeding. In addition to VPA, 34 patients (63.0%) received an average of 1.8 drugs, the most common being benzodiazepines (12 cases), followed by clobazam (10 cases). The underlying diseases mainly include neonatal asphyxia (13 cases), congenital anomalies of the central nervous system (10 cases), chromosomal disease (9 cases), secondary epilepsy (11 cases) and idiopathic epilepsy (11 cases). Six patients (28%) were receiving or had received carnitine supplements.

**Table 1:** Basic information of the 54 patients included.

**Parameter**		**value**
Sex	males	28 (51.9%)
	females	24 (44.4%)
	Na	2 (3.7%)
Age	Years	7 (2, 34) ^b^
	2–17	50 (92.6%)
	19–34	4 (7.4%)
Country	Japan	28 (51.9%)
	Spain	7 (13.0%)
	USA	9 (16.7%)
	UK	8 (14.8%)
	Canada	1 (1.9%)
	Iran	1 (1.9%)
Severe disability	Yes	47 (87.0%)
	No	7 (13.0%)
Other AEDs (34)^a^	benzodiazepine	12 (29.6%)
	clobazam	10 (29.4%)
	topiramate	6 (17.6%)
	carbamazepine	5 (14.7%)
	phenobarbital	5 (14.7%)
	zonisamide	4 (11.8%)
	phenytoin	3 (8.8%)
	average number of drugs	1.8
	1 drug	14 (41.2 %)
	2 drugs	14 (41.2 %)
	3 drugs	4 (11.8%)
	4 drugs	2 (5.9%)
Clinical remarks	neonatal asphyxia	13 (24.1%)
	congenital anomalies of the central nervous system	10 (18.5%)
	chromosomal disease	9 (16.7%)
	secondary epilepsy	11 (20.4%)
	idiopathic epilepsy	11 (20.4%)

UK, United Kingdom; AEDs, antiepileptic drugs.

a Represents the number of patients out of 54 for whom information regarding this particular parameter was provided.

b Median (minimum-maximum).

### Administration of sodium valproate

The median duration of VPA treatment before FS was 4 years (range 0.7–15.5) (Table 2). The median dose at diagnosis was 35 mg/kg/day (range 14–70) in 20 patients. Plasma concentrations were measured in 27 patients during VPA treatment. The median plasma concentration of VPA was 77.6 μg/ml (range 34–142), which was lower than the recommended value in four patients (14.8%) and higher than the recommended value in six patients (22.2%).

**Table 2:** Clinical characteristics and administration of valproate in the 54 included patients.

**Parameter**		**Value**
Duration (44)^a^	Years	4 (0.7,15.5)^b^
VPA levels (27)^a^	μg/ml	74.2 (34, 142)^b^
	<50	4 (14.8)
	50–100	17 (63.0%)
	>100	6 (22.2%)
VPA dose (20)^a^	mg/kg/day	35 (14, 70)^b^
Route of administration	tube feeding	35(64.8%)
	orally	3 (5.6%)
	Na	16 (29.6%)
Clinical manifestations	fractures	14 (25.9%)
	osteoporosis	4 (7.4%)
	rickets	3 (5.6%)
	fever	6 (11.1%)
	muscle weakness	5 (9.3%)
	edema	5 (9.3%)
	drowsiness	3 (5.6%)
	vomiting	2 (3.7%)
	polyuria	2 (3.7%)
	alopecia	1 (1.9%)
	weight gain	1 (1.9%)
	weight loss	1 (1.9%)
	routine screening tests	18 (33.3%)

VPA, Valproic acid; Na, not applicable.

a Represents the number of patients out of 54 for whom information regarding this particular parameter was provided.

b Median (minimum-maximum).

### Clinical characteristics

The clinical characteristics of the 54 patients are summarized in Table 3. Fractures occurred in 14 patients (25.9%) and rickets in four patients (7.4%). Six patients (11.1%) had unexplained fever in primary selection, and five patients (9.3%) had muscle weakness and edema. Eighteen patients (33.3%) with FS were found only in incidental laboratory tests and routine screening tests.

**Table 3:** Serum biochemical analysis of 54 patients included.

**Parameter**		**Value**
*P* (50)^a^	normal	1 (2.0%)
4.7–6.7 mg/dl	hypophosphatemia	49 (98.0%) 1.2 (0.3, 3.2)^b^
Na+ (25)^a^	normal 1	14 (56.0%)
135–145 mmol/L	hyponatremia	3 (12.0%) 133 (125, 133)^b^
	hypernatremia	6 (24.0%) 155 (146, 202)^b^
K (39)^a^	normal	12 (30.8%)
3.5–5.0 mmol/L	hypokalemia	27 (69.2%) 3 (1.4, 3.4)^b^
Ca (18)^a^	normal	14 (77.8%)
8.4–10.4 mg/dl	hypocalcemia	4 (22.2%) 7.9 (7.6, 8.2)^b^
Cl (17)^a^	normal	6 (35.3%)
100–108 mmol/L	hypochloremia	1 (5.9%)
	hyperchloremia	10 (58.8%) 116 (110, 140)^b^
BUN (23)^a^	normal	20 (87.0%)
6–21 mg/dl	elevated	3 (13.0%) 28.9 (22.4, 30)^b^
Cr (24)^a^	normal	17 (70.8%)
0.6–1.1 mg/dl	elevated	5 (20.8%) 1.39 (1.12, 1.7)^b^
UA (30)^a^	normal	2 (6.7%)
2.0–7.0 mg/dl	<2	28 (93.3%) 0.8 (0.4, 1.94)^b^
HCO_3_ (44)^a^	normal	6 (13.6%)
22–29 mmol/L	<22	35 (79.5%) 17(5.2, 21.8)
	>29	3 (6.8%) 30.6(29.3, 36.1)^b^
pH (32)^a^	normal	6 (18.8%)
7.350–7.450	<7.35	26 (81.2%) 7.262 (6.98, 7.336)^b^
Carnitine (18)^a^^#^	normal	10 (55.6%)
22–66 mmol/L	hypocarnitinemia	8 (44.4%) 16.8 (5.7, 20.1)^b^

Na, sodium; K, potassium; Cl, chloride; P, phosphate; Ca, calcium; Cr, Creatinine; BUN, blood urea nitrogen; UA, uric acid.

# free carnitine.

a Represents the number of patients out of 54 for whom information regarding this particular parameter was provided.

b Median (minimum-maximum).

### Laboratory test

Serum biochemistry and urinalysis of FS patients are summarized in Table 3. Laboratory results at the onset of Fanconi syndrome showed hypophosphatemia in 49 patients (98.0%) with a median level of 1.2 mg/dl (range 0.3–3.2), hypokalemia occurred in 27 patients (69.2%) with a median level of 3 mmol/L (range 1.4–3.4), hypocalcemia occurred in four patients (22.2%) with a median level of 7.9 mg/dl (range 7.6–8.2), hyperchloremia occurred in 10 patients (58.8%) with a median level of 116 mmol/L (range 110–140), hypernatremia occurred in six patients and hyponatremia in three patients, with median levels of 155 mmol/L (range 146–202) and 133 mmol/L (range 125–133), respectively. Five patients (20.8%) presented with elevated creatinine in the 24 patients examined, with a median level of 1.39 mg/dl (range 1.12–1.7). Three patients (13.0%) presented with elevated blood urea nitrogen in the 23 patients examined, with a median level of 28.9 mg/dl (range 22.4–30). Twenty-eight patients (93.3%) developed hypouricemia with a median level of 0.8 mg/dl (range 0.4–1.94). Blood gas analysis showed that 26 patients (81.2%) had low blood pH, with a median level of 7.262 (range 6.98–7.336). Of the 18 patients whose serum carnitine levels were described, eight patients (44.4%) developed hypocarnitineemia.

The urinalysis of 54 FS patients is summarized in Table 4. Urinalysis revealed glycosuria in 49 patients (96.1%), proteinuria in 47 patients (100.0%), generalized hyperaminoaciduria in 26 patients (100.0%), and urine pH higher than eight in two patients (7.7%). The median level of β2-MG in the 30 patients was 52.96 mg/L (range 1.66–143). Thirty-two patients (94.1%) had a reduction in %TRP, with a median level of 65.5% (range 0–79.9%). Twenty-three patients (100%) had elevated FEUA, with a median level of 52.8% (range 16.4–104.9%).

**Table 4:** Urinalysis, renal biopsy, and ultrasonography of the 54 included patients.

**Parameter**		**Value**
Urine analysis		
pH (26)^a^	>8	2 (7.7%)
5.0–8.0	normal	24 (92.3%)
glucose (51)^a^	glucosuria	49 (96.1%)
	negative	2 (3.9%)
Protein (47)^a^	proteinuria	47 (100.0%)
Amino acid (26)^a^	hyperaminoaciduria	26 (100.0%)
β2- MG (30)^a^ 0–0.2 mg/L		30 (100.0%) 52.96 (1.66,143)^b^
%TRP (34)^a^	normal	2 (5.9%)
80–94%	reduced	32 (94.1%) 65.5% (0, 79.9%)
%FEK (12)^a^	elevated	4 (33.3%)
5.7–16.5%	reduced	2 (16.7%)
	normal	6 (50.0%)
% FEUA (23)^a^	elevated	23 (100%)
4–14%		52.8% (16.4, 104.9%)
FENa (3)^a^ <1.0%	elevated	3 (100%) 1.5% (1.5, 4%)
Renal ultrasound (12)^a^	normal	6 (50.0%)
	nephrocalcinosis	2 (16.7%)
	hyperechogenicity	4 (33.3%)
Renal biopsy (5)^a^	interstitial nephritis	3 (60.0%)
	immuno-deposition	2 (10.0%)
	infiltration of CD4 positive cells	2 (40.0%)
	interstitial fibrosis	1(20.0%)
	giant mitochondria in the proximal tubular cells, and abnormal round granular inclusions	1 (20.0%)
	decreased expression of proximal tubule markers and increased expression of markers of distal nephron differentiation	1 (20.0%)

β2 MG, β2 macroglobulin; FEK, fractional excretion of potassium; TRP, percent total reabsorption of phosphate; FEUA, fractional excretion of uric acid; FENa, fractional excretion of sodium.

a Represents the number of patients out of 54 for whom information regarding this particular parameter was provided.

b Median (minimum-maximum).

### Imaging tests and kidney biopsy

Renal ultrasonography and pathological analysis are summarized in Table 4. Nephrocalcinosis (2 cases) and hyperechoic (4 cases) were present in 12 patients who underwent renal ultrasonography. Kidney biopsies were performed in five patients, mainly showing interstitial nephritis (3 cases) with or without immune deposition, CD4-positive cell infiltration and interstitial fibrosis.

### Treatment and prognosis

Treatment and prognosis of 54 patients are summarized in Table 5. Forty-eight patients (88.9%) were discontinuing VPA, and one patient (1.9%) had a VPA dose reduction. Seven patients (13.0%) received carnitine supplementation. Twenty-two patients (40.7%) were given supplemental therapy such as citrate, phosphate, potassium. Fifty-two patients (96.3%) had complete recovery from FS in a median of 3 months (range 0.25–18). One patient had persistent proteinuria and glycosuria after discontinuation of VPA, and one patient had a further increase in creatinine after slow dose reduction. Twenty-one patients (67.7%) exhibited complete FS, while 10 patients (32.3%) exhibited incomplete FS.

**Table 5:** Treatment and prognosis of the 54 included patients.

**Parameter**		**Value**
Treatment	valproate	
	discontinuation	48(88.9%)
	lower dose	1 (1.9%)
	na	5 (9.3%)
	carnitine	7 (13.0%)
	supplementation treatment: citrate, phosphate, potassium, bicarbonate, hydration, Vitamin D	22 (40.7%)
Prognosis	recovery	52 (96.3%)
	persistent aminoaciduria and glucosuria	1 (1.9%)
	increased creatinine	1 (1.9%)
Time until recovery (42)^a^	months	3 (0.25,18) ^b^
Type of Fanconi syndrome (31)^a^	complete	21 (67.7%)
	incomplete	10 (32.3%)

Na, not applicable.

a Represents the number of patients out of 54 for whom information regarding this particular parameter was provided.

b Median (minimum-maximum).

## Discussion

The number of reports of VPA-induced FS has been increasing since the first report. Although most VPA -induced FS originate from Asia. It is unclear whether geographic or ethnic background plays a role in pathogenesis. There was no clear relationship between VPA-induced FS and VPA dose or duration of treatment, occurring at least at 8 months. Except for four adult patients, the remaining patients were children, and most were tube fed and severely disabled. For people with epilepsy, a combination of different antiepileptic drugs may be required. Most patients received an average of two antiepileptic drugs other than VPA at the time of diagnosis of FS. VPA combined with other antiepileptic drug therapy may be a risk factor for FS. There is no conclusive evidence that topiramate and other AEDs can induce FS. Nevertheless, concomitant use of topiramate exacerbates metabolic acidosis in these patients and may produce more severe bone changes that increase the risk of microfractures (11). But further research may be needed on which antiepileptic drug to combine. Consistent with our study, Watanabe et al. demonstrated that young age, severe disability from tube feeding, and multiple drug treatments for seizures were contributing factors (12). In addition, epilepsy itself may also be a cause of renal tubular dysfunction (13). We should note that CDKL5 heterozygous mutations in two patients can lead to mitochondrial dysfunction, which may predispose patients to renal tubular damage after exposure to VPA (14, 15).

In our experience, the dose and duration of valproate was not associated with the severity and completeness of FS. More than 90% of the patients in this study developed hypophosphatemia (98.0%), hypouricemia (93.3%), phosphate urine (94.1%), glycosuria (96.1%), proteinuria (100.0%), hyperaminoaciduria (100.0%), urine β2-MG (100.0%). Some patients were free of metabolic acidosis (18.8%) and hypokalemia (30.8%). A possible explanation for the hypochloremia in one patient was hypochloremia is occasionally found in patients with anion gap acidosis but without exposure to a recognized alkalosis-inducing process (16). Therefore, serum phosphate, serum uric acid, proteinuria, glycosuria, hyperaminoaciduria, and urinary β2-MG levels should be used as screening tests to detect VPA-induced FS. Most patients with VPA-induced FS are asymptomatic, with hypophosphatemia, hypouricemia, elevated urinary β2-MG, and generalized hyperaminoaciduria detected only on routine screening tests or incidental laboratory studies. Therefore, routine screening of urinalysis and serum electrolyte levels in patients receiving VPA may reveal more patients with FS. Although renal calcium loss is part of the proximal tubulopathy of FS, nephrocalcinosis is not a common feature of patients due to the concurrent excessive loss of citrate in the urine, patients also presented also with this condition, and the concomitant use of topiramate might have potentiated this condition (17, 18). Topiramate promotes intrarenal calcium deposition and nephrocalcinosis by causing increased bicarbonate loss and increased urinary pH and hypocitrate (19).

Some patients present with osteoporosis, rickets, or fractures by imaging. If FS is not diagnosed early, patients may develop pathological fractures. What will be done in the future is to assess which patients may be more likely to develop renal tubular disease and bone mineralization effects after taking the drug. Delays in diagnosis may lead to invasive tests, such as renal biopsy, although there is no benefit in the diagnosis of FS. These patients had no previous renal dysfunction and severe renal tubular disease, and renal biopsy was performed to confirm the cause of glomerular hypofunction and/or renal tubular dysfunction. VPA should be considered to be a potential cause of interstitial nephritis resulting in FS in epileptic children (12, 20, 21). Drug-induced interstitial nephritis is caused by drug-dose-independent hypersensitivity reactions. Of the three patients with valproic acid-induced interstitial nephritis, hypersensitivity to VPA was proved to be present in one patient by the results of both a skin patch test and the release of TNF-β (20). The other two patients could not prove hypersensitivity to VPA, and one patient developed unexplained fever, but the blood concentration of valproic acid was higher than the normal range, and direct toxic effects could not be ruled out (12, 21). Extrarenal manifestations of hypersensitivity are associated with fever, rash, mild arthralgia, and eosinophilia (22). FS secondary to VPA should be considered when unexplained fever occurs in individuals taking VPA (12, 23, 24).

VPA is associated with reduced carnitine levels and occasionally with true carnitine deficiency (25). An *in vitro* study by Heidari et al. found that high-dose carnitine (100 mg/kg/d) attenuated VPA-induced renal dysfunction and alleviated mitochondrial energy disturbances (26). Ono et al. reported that maintaining normal serum carnitine levels was not always effective in preventing VPA-induced FS (27). This requires more research to further demonstrate the role of serum carnitine in FS.

The exact pathogenesis of VPA-induced FS has not been elucidated. It may be associated with mitochondrial defects secondary to treatment-related carnitine deficiency. Mitochondrial dysfunction and oxidative stress contributed to the VPA-induced FS (26). Another speculation is that it interacts with cleavage of fibroblast growth factor 23 (FGF23), resulting in high FGF23 levels and renal phosphate loss (28). Tubulointerstitial nephritis due to hypersensitivity to VPA or the direct toxic effects of VPA, which is often accompanied by systemic allergic manifestations including rash and fever (12, 29). VPA may interact with sodium-dependent cotransporters NaPi-IIa on the apical membrane, or a Na/K ATPase pump on the basolateral membrane (30, 31). Further studies are needed to determine the pathophysiological mechanism by which VPA induced FS.

The mainstay of treatment for FS secondary to VPA is valproate discontinuation. In addition, renal solute loss and concomitant bone disease should be treated (1). Hypophosphatemia should be treated with 1 to 3 g/day of oral phosphate, and hypokalemia requires potassium supplementation, especially in the presence of significant renal tubular acidosis. Many people with Fanconi syndrome require vitamin D supplementation to treat rickets and osteomalacia. The evidence for carnitine supplementation to prevent or treat VPA-induced FS is insufficient. Hyperaminoaciduria, glycosuria, proteinuria, and hyperuricuria do not require specific treatment. VPA-induced FS appears to be reversible after discontinuation, with abnormal laboratory values subsiding within variable times, although hypouricemia persisted in some individuals.

## Conclusion

Clinicians should be aware that FS is a consequence of long-term VPA use, especially in patients who are younger, tube-fed, severely disabled, and combined with anticonvulsants. Serum electrolytes, serum phosphate, uric acid, and urinalysis should be performed regularly in patients receiving VPA.

## Data availability statement

The original contributions presented in the study are included in the article/supplementary material, further inquiries can be directed to the corresponding author.

## Author contributions

CW and WF: conception and design. CW, YZ, LS, and ZD: data collection, methodology, analysis, data interpretation, data curation, statistical analysis, and revision. All authors: manuscript writing and final approval of manuscript.

## Funding

This study was supported by research grants from the National Natural Science Foundation of China (81900344).

## Conflict of interest

The authors declare that the research was conducted in the absence of any commercial or financial relationships that could be construed as a potential conflict of interest.

## Publisher's note

All claims expressed in this article are solely those of the authors and do not necessarily represent those of their affiliated organizations, or those of the publisher, the editors and the reviewers. Any product that may be evaluated in this article, or claim that may be made by its manufacturer, is not guaranteed or endorsed by the publisher.
